# Transcriptomic Analysis of *Hippocampus abdominalis* Larvae Under High Temperature Stress

**DOI:** 10.3390/genes15101345

**Published:** 2024-10-21

**Authors:** Wenjie Xiao, Baoying Guo, Jie Tan, Changlin Liu, Da Jiang, Hao Yu, Zhen Geng

**Affiliations:** 1 Yellow Sea Fisheries Research Institute, Chinese Academy of Fishery Sciences, Qingdao 266071, China; 13626138130@163.com (W.X.); tanjie@ysfri.ac.cn (J.T.); kdajiavg@163.com (D.J.); 15106527336@163.com (H.Y.); weishouyong2024@163.com (Z.G.); 2 Marine Science and Technology College, Zhejiang Ocean University, Zhoushan 316022, China; guobaobao2000@126.com

**Keywords:** *Hippocampus abdominalis*, larvae, temperature stress, transcriptome, RT-qPCR

## Abstract

Objectives: Acute temperature stress was explored in *Hippocampus abdominalis* through a comprehensive RNA-seq analysis. Methods: RNA-seq was conducted on 20-day-old *H. abdominalis* after 24 h of temperature stress. Four experimental conditions were established: a control group (18 °C) and three temperature treatment groups (21, 24, and 27 °C). Results: Seahorse larvae were found to be unaffected by 21 °C and 24 °C and were able to survive for short periods of time during 24 h of incubation, whereas mortality approached 50% at 27 °C. The sequencing process produced 75.63 Gb of high-quality clean data, with Q20 and Q30 base percentages surpassing 98% and 96%, respectively. A total of 141, 333, and 1598 differentially expressed genes were identified in the 21, 24, and 27 °C groups vs. a control comparison group, respectively. Notably, the number of up-regulated genes was consistently higher than that of down-regulated genes across all comparisons. Gene Ontology functional annotation revealed that differentially expressed genes were predominantly associated with metabolic processes, redox reactions, and biosynthetic functions. In-depth KEGG pathway enrichment analysis demonstrated that down-regulated genes were significantly enriched in pathways related to steroid biosynthesis, terpenoid backbone biosynthesis, spliceosome function, and DNA replication. Up-regulated genes were enriched in pathways associated with the FoxO signaling pathway and mitophagy (animal). The results indicated that temperature stress induced extensive changes in gene expression in *H. abdominalis*, involving crucial biological processes such as growth, biosynthesis, and energy metabolism. Conclusions: This study provided key molecular mechanisms in the response of *H. abdominalis* to temperature stress, offering a strong basis for future research aimed at understanding and mitigating the effects of environmental stressors on marine species.

## 1. Introduction

Aquatic ecosystems face a variety of complex environmental stressors that interact with each other and pose serious challenges to the survival and reproduction of organisms in them. Climate change, as one of the most significant environmental stressors, profoundly alters the living environment of aquatic organisms by affecting water temperature, acidity, salinity, and other aspects [1]. Among them, temperature, as a crucial environmental variable, has a direct and far-reaching impact on the physiological functions, behavioral habits, and population dynamics of fish [2]. Studies have shown that small changes in temperature are sufficient to trigger adjustments in the metabolic rate of fish, which in turn affects their food requirements, growth rates, and reproductive strategies [3]. More seriously, when water temperature exceeds the physiological tolerance range of fish, it can lead to thermal stress, including reduced immunity, reduced fecundity, and even outright mortality [4]. Especially in the context of global warming, extreme heat events are occurring frequently, posing an unprecedented existential threat to fish and other aquatic organisms.

*H. abdominalis* is classified within the Gasterosteiformes order, Syngnathidae family, and Hippocampus genus. It is commonly found in the shallow coastal waters of tropical, subtropical, and temperate regions, with a primary distribution across the South Pacific, particularly around the coasts of Australia and New Zealand. This species typically inhabits areas with seagrass or macroalgae [5]. With the increase in global warming, the high temperature in summer lasts too long, which seriously affects the survival rate of seahorses and jeopardizes the economic benefits of farmers. Research on the effects of temperature on *H. abdominalis* is currently limited. Woods identified a temperature tolerance range of 8 to 24 °C in the wild [6], while Luo et al. found that the optimal aquaculture temperature for this species is 16 °C based on survival and growth metrics [5]. However, there has been no reported research on the molecular mechanisms underlying temperature effects on *H. abdominalis*.

RNA sequencing (RNA-seq) is a powerful technique that operates independently of genomic sequences, allowing for the direct sequencing of complementary DNA (cDNA). This method provides a robust means to measure gene expression levels, identify and analyze differentially expressed genes, and explore the mechanisms of gene expression regulation [7,8]. In recent years, transcriptome sequencing has played a pivotal role in elucidating the molecular mechanisms underlying biological responses to environmental stressors. For instance, Yang et al. used transcriptome sequencing to analyze kidney tissues from European turbot (*Scophthalmus maximus*) subjected to various high-temperature treatments [9]. Their work led to the creation of a comprehensive heat-stress kidney transcriptome database, which serves as a valuable resource for breeding programs aimed at developing heat-resistant aquaculture strains. Additionally, Yang et al. used transcriptome sequencing to investigate the effects of acute cold stress on energy metabolism and immune regulation in *Macrobrachium rosenbergii* [10]. Their study identified key pathways and genes involved in cold stress responses, providing critical insights into the molecular mechanisms of low-temperature adaptation. Wang et al. conducted transcriptome analyses of cuttlefish larvae exposed to high temperatures and found that a large number of DEGs play key roles in the stress response and cause severe damage to the stress system, providing insights into the intrinsic mechanisms of high-temperature stress in cephalopods [11]. In the present study, we used the Illumina sequencing platform to conduct transcriptome sequencing on *H. abdominalis* larvae subjected to temperature stress. This research focused on identifying and characterizing the relevant functional genes involved in temperature stress responses and elucidating the regulatory mechanisms governing these responses. By examining the transcriptomic profiles of these larvae under varying temperature conditions, we aimed to uncover the molecular mechanisms through which temperature stress affected their development and physiology. Our results are expected to offer valuable theoretical insights and empirical data that will support the advancement of aquaculture practices and the development of temperature-resistant strains, thereby contributing to the broader field of marine biology and aquaculture research.

## 2. Materials and Methods

### 2.1. Experimental Materials

For this study, a cohort of 20-day *H. abdominalis* was selected based on uniform size and robust health. These seahorses, with an average body weight of 0.049 ± 0.005 g and an average body length of 2.5 ± 0.3 cm, were supplied by Qingdao Qingyuan Marine Biotechnology Co., Ltd. (Qingdao, China). The seahorses were maintained in a 3 cubic meter rearing tank, with a water depth of 1 m. The tank was equipped with continuous aeration to ensure adequate oxygen levels and regular maintenance was carried out, including the removal of uneaten food, fecal matter, and any deceased individuals to preserve water quality.

Feeding occurred twice daily, once at 9:00 AM and again at 4:00 PM, with brine shrimp (Artemia) nauplii provided as the primary food source. The density of the Artemia was carefully regulated at five to six individuals per milliliter to meet the nutritional needs of the seahorses. Throughout the experimental period, environmental conditions in the tank were closely monitored and maintained: the water temperature was kept stable at 18 ± 0.2 °C, the salinity was maintained at 30 ± 0.1 ppt, the pH level was controlled at 7.9 ± 0.2, and the dissolved oxygen (DO) concentration was consistently held at 6 mg/L or above. These carefully regulated conditions were crucial for ensuring the optimal health and development of the seahorses during this study.

### 2.2. Experimental Method

The experimental setup comprised three temperature gradients: 21, 24, and 27 °C, designated as A21, B24, and C27, respectively. A control group was maintained at 18 °C, designated as D18. Each temperature condition was replicated in three parallel tanks, with 30 seahorses per tank, resulting in a total of 360 seahorses for this study. At the start of the experiment, the seahorses were rapidly transferred from the holding tank at 18 °C to rearing tanks that had been pre-adjusted to the designated experimental temperatures. Temperature control within the tanks was maintained using heating rods throughout the experiment. The experimental culture tanks had a volume of 36 × 28 × 24 cm^3^ and 10 L of water was placed in each tank.

During the experimental period, feeding was conducted as usual, and 1/3 of the tank water was replaced every 6 h to maintain water quality. After 24 h of temperature stress, three seahorse larvae were randomly sampled from each tank and immediately placed into pre-labeled cryovials. These samples were then rapidly frozen in liquid nitrogen and subsequently stored in a −80 °C for subsequent transcriptome analysis.

### 2.3. Transcriptome Library Construction and Sequencing

Total RNA was extracted from puffed seahorse larvae of experimental and control groups using a TIANGEN-brand total RNA extraction kit according to the instructions, and the concentration and quality of the extracted total RNA were detected using a NanoDrop microspectrophotometer and 1% agarose gel electrophoresis, respectively. For each group, RNA was obtained from three pooled individuals, which were homogenized and processed. The integrity of the RNA samples was assessed using an Agilent 2100 Bioanalyzer (Agilent Technologies, Santa Clara, CA, USA) to ensure an accurate evaluation of RNA quality.

Following extraction, cDNA fragments of approximately 250–300 base pairs (bp) in length were selected using AMPure XP beads (Beckman Coulter, Brea, CA, USA). These selected fragments underwent PCR amplification, and the resulting PCR products were purified to create the final cDNA library. Once the library quality was verified, sequencing was performed using the Illumina platform. Optical signals were converted into sequencing peaks with the help of specialized computer software, providing the sequence information for the target fragments.

### 2.4. Differentially Expressed Gene Analysis and Functional Annotation

The initial step involved filtering raw sequencing data to eliminate reads with adapters, ambiguous bases, and low-quality regions, resulting in clean data. These clean data were then aligned to the reference genome (https://ngdc.cncb.ac.cn/gwh/Assembly/18745/show, accessed on 2 November 2023) to generate mapped data. Following alignment, the quality of the library was assessed, and structural-level analyses were performed.

Subsequently, differential expression analysis was conducted to identify differentially expressed genes (DEGs) among the various temperature groups. Functional annotation of DEGs was carried out using Gene Ontology (GO) and Kyoto Encyclopedia of Genes and Genomes (KEGG) pathway enrichment analyses. Differential gene expression was assessed using DESeq2 (1.20.0), with the significance of differential expression determined by a threshold of |log2(Fold Change)| ≥ 1 and padj ≤ 0.05. A lower padj value reflects a more pronounced differential expression. clusterProfiler software (3.8.1) was used for GO and KEGG enrichment analyses, with a significance threshold set at padj < 0.05 to identify significantly enriched pathways and functions.

### 2.5. Verification by Quantitative Real-Time PCR

From the identified DEGs, a selection of 11 genes was chosen for further analysis. Primers for these genes were designed using Primer Premier 5 software (Table 1). Quantitative PCR (qPCR) was performed with *ap4b1* as the internal reference gene. Each gene was analyzed in triplicate for all samples. Relative expression levels were quantified using the 2^−ΔΔCt^ method.

## 3. Results

### 3.1. Transcriptome Sequencing Basic Data

For the juvenile seahorse experimental groups (A21, B24, and C27) and the control group (D18), we compiled a comprehensive dataset consisting of 75.63 Gb of clean data following rigorous quality control procedures. Each sample achieved a minimum of 5.79 Gb of clean data. An in-depth analysis of base quality and composition revealed that the Q30 percentage, an indicator of sequencing accuracy, was consistently above 96.34% for all samples. Additionally, the GC content across the samples varied between 51.29% and 53.35%, indicating a well-distributed base composition. Detailed sequencing metrics for each sample are summarized in Table 2.

The clean reads obtained were aligned with the reference seahorse genome. This resulted in an impressive overall alignment rate exceeding 88.21% and a unique alignment rate of over 83.83%. These alignment statistics are detailed in Table 3.

Further analysis of the alignment results indicated that a significant majority of the clean reads were mapped to exonic regions of the genome, with percentages ranging from 82.63% to 85.40%. In contrast, only a small fraction of reads, between 3.09% and 4.35%, were mapped to intronic regions, while 11.51% to 13.67% of reads were mapped to intergenic regions. The distribution of sequencing reads across various genomic regions for all samples is presented in Table 4.

Overall, these results demonstrated that the transcriptome sequencing data were of high quality, meeting the necessary criteria for further in-depth analysis. The robustness and completeness of the dataset provided a solid foundation for subsequent research and interpretations.

### 3.2. Transcriptome Sequencing of Differentially Expressed Genes

**Figure 1:**
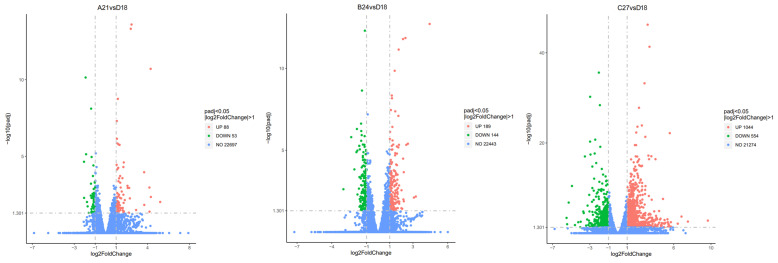
Volcano map of differentially expressed genes in *H. abdominalis* larvae.

To investigate the effects of high temperature stress on gene expression, we conducted a comprehensive analysis of DEGs across various temperature conditions. The data are visualized in Figure 1. The comparison between the A21 and D18 groups identified a total of 141 DEGs, with 88 genes up-regulated and 53 genes down-regulated in response to the temperature shift. In the comparison between B24 and D18, we detected 333 DEGs, consisting of 189 up-regulated and 144 down-regulated genes. The most pronounced temperature shift occurred between the C27 vs. D18 groups, which resulted in 1598 DEGs, including 1044 up-regulated and 554 down-regulated genes.

**Figure 2:**
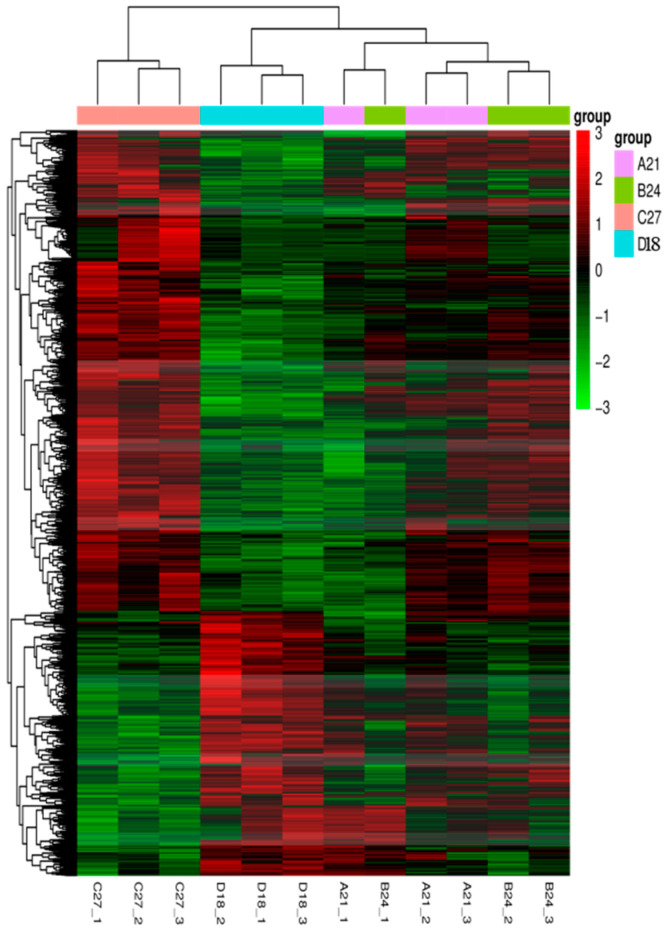
Clustering heat map of differentially expressed genes.

**Figure 3:**
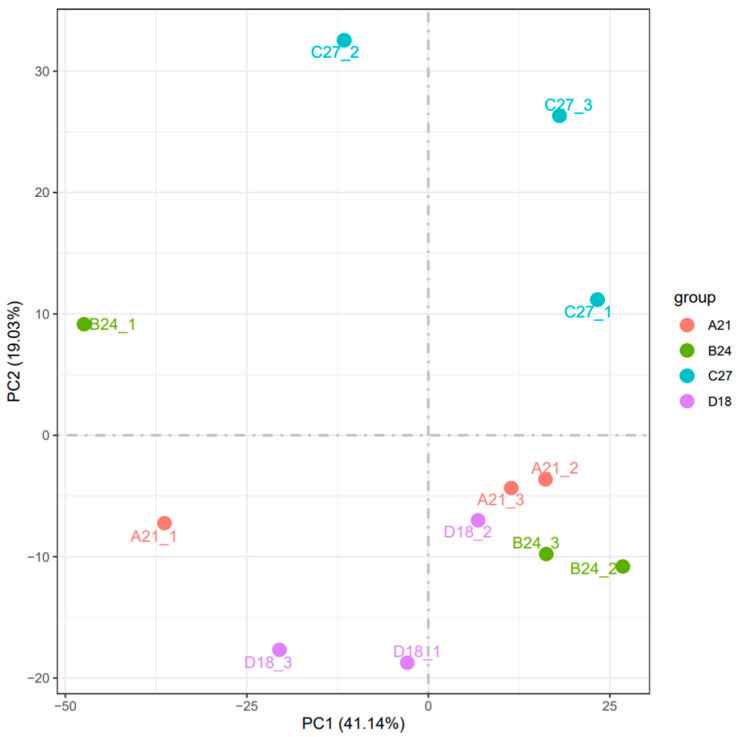
Principal component analysis diagram.

The clustering analysis of these DEGs is illustrated in Figure 2. Samples within each temperature group were well clustered together, suggesting minimal variability within groups and significant differentiation between groups. Heatmaps of differentially expressed gene clustering in transcriptomes not only demonstrate differences in gene expression, but also reveal common expression patterns of functional genes. Good clustering of samples within a group may imply that these samples share a high degree of similarity in the expression of functional genes, which may share a common involvement in some important physiological process or metabolic pathway of the organisms, leading to similar phenotypic features. This observation was further corroborated by the principal component analysis (PCA) depicted in Figure 3. The PCA results demonstrated a clear separation of samples across different temperature conditions. A notable similarity occurred between the control group (D18) and the 21 °C group (A21), whereas other temperature groups (B24 and C27) exhibited more distinct separation.

**Figure 4:**
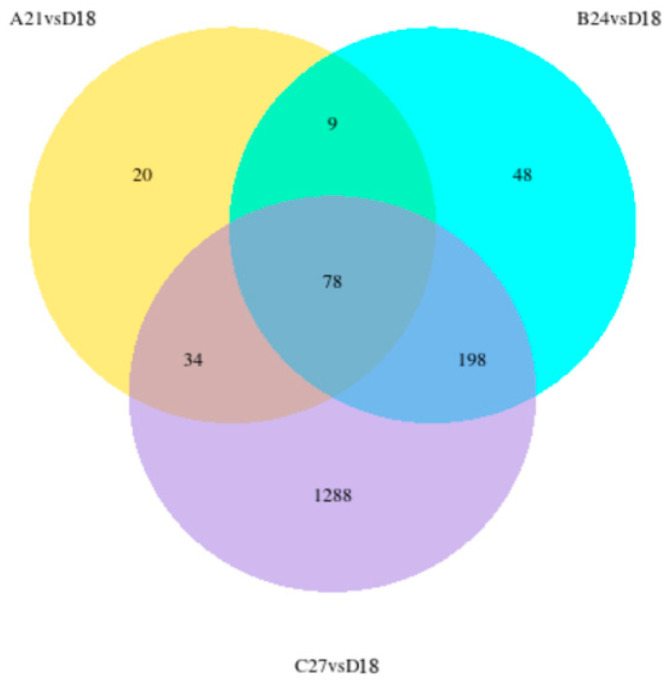
Venn diagram of differentially expressed genes in the transcriptome.

The differences in gene expression between each temperature stress group and the control group can be visualized and are effectively shown using a Venn diagram (Figure 4). There were 87 (78 + 9) common differential genes between the A21 vs. D18 group and the B24 vs. D18 group; 276 (78 + 198) common differential genes between the B24 vs. D18 group and the C27 vs. D18 group; and 112 (34 + 78) common differential genes between the A21 vs. D18 group and the C27 vs. D18 group. A total of 78 genes are shared across all comparison groups. The number of unique genes expressed after stress was 20 in the A21 vs. D18 group, 48 in the B24 vs. D18 group, and 1288 in the C27 vs. D18 group.

### 3.3. GO Enrichment Analysis

**Figure 5:**
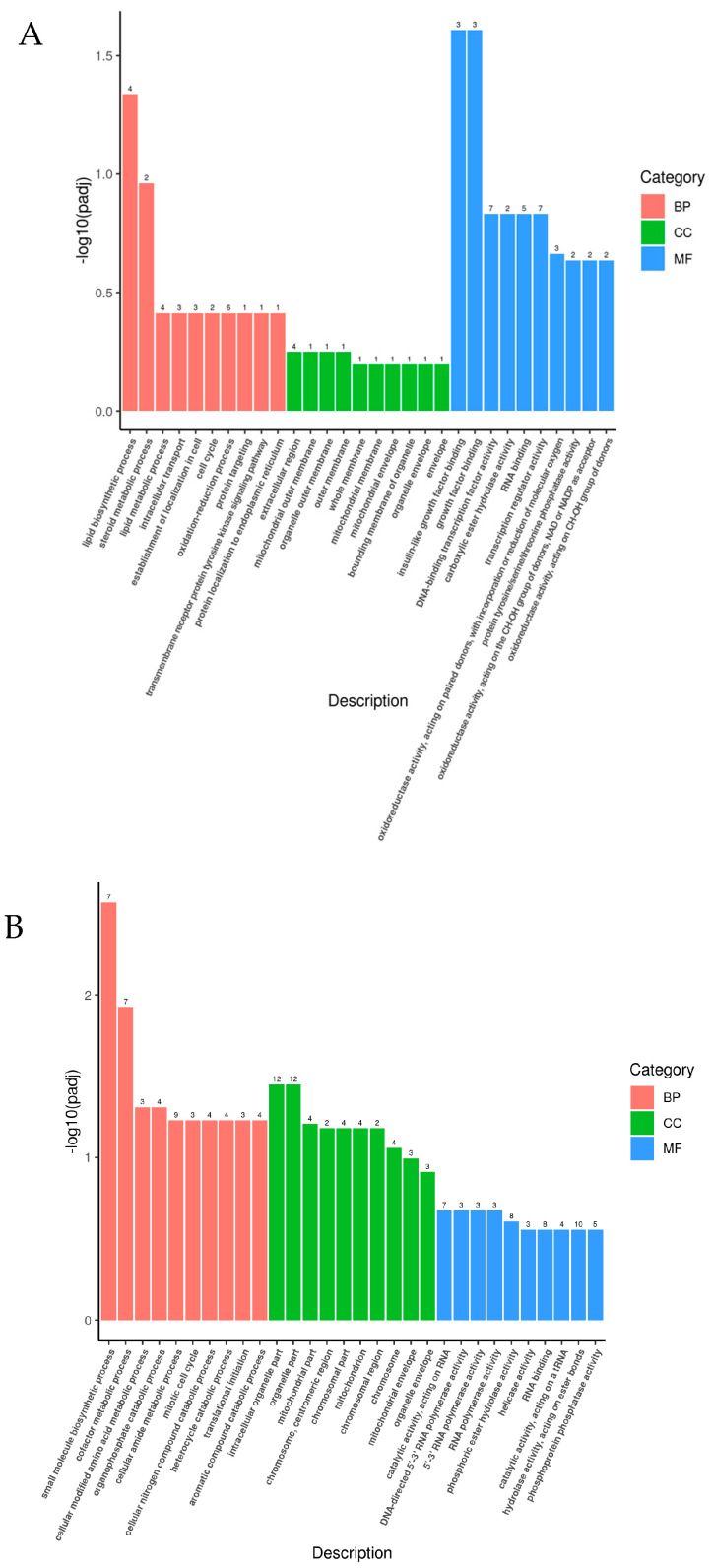

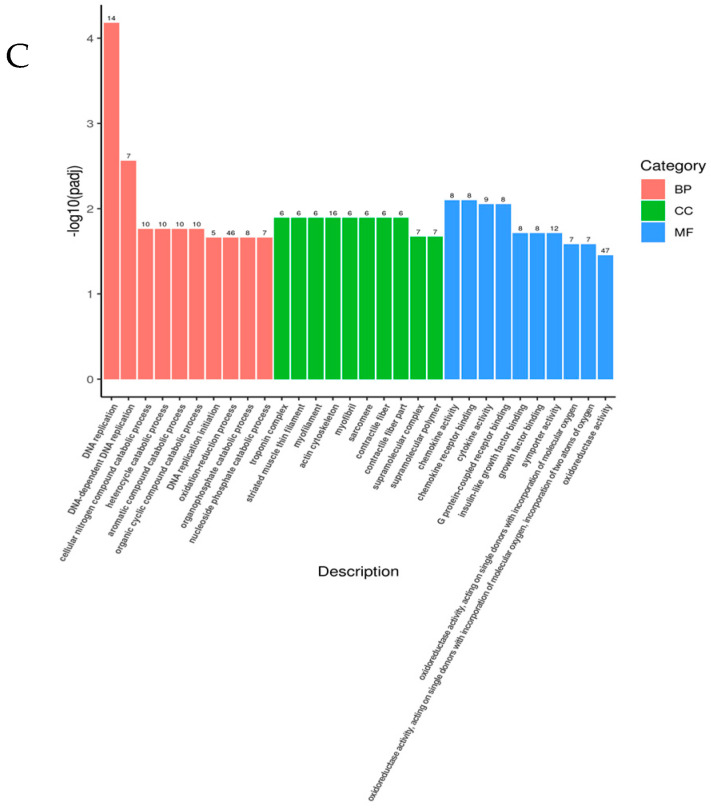
GO enrichment analysis of transcriptome differentially expressed genes. (**A**): A21 vs. D18; (**B**): B24 vs. D18; (**C**): C27 vs. D18. In the figure, the horizontal coordinate is the GO term, and the vertical coordinate is the significance level of the GO term enrichment expressed as −log10 (padj). Different colors represent different functional classifications.

The results of GO enrichment analysis showed that DEGs were distributed in three categories: biological process, cellular component, and molecular function. Figure 5 shows the results of the GO enrichment analysis of DEGs between different temperature-treated groups and the control group at 24 h of stress.

In the A21 vs. D18 group, there was a significant GO enrichment in biological processes and molecular functions (padj < 0.05). In biological processes, the significantly enriched GO term was the lipid biosynthetic process; in molecular function, significantly enriched GO terms were related to growth factor binding (Figure 5A).

Significant GO enrichment (padj < 0.05) was observed in the B24 vs. D18 group in biological processes and cellular compositional levels. The terms small molecule biosynthetic process, cofactor metabolic process, cellular modified amino acid metabolic process, and organophosphate catabolic process were significantly enriched in biological processes. In the cellular fractions, the two GO terms with the most differentially annotated genes were both related to organelle parts, and both annotated 12 differentiated genes (Figure 5B).

In the C27 vs. D18 group, there was significant GO enrichment in all three levels (padj < 0.05). Among biological processes, the significantly enriched GO term was related to DNA replication, and the GO term with the most enriched differential genes was the oxidation-reduction process, with 46 differential genes annotated. Among cellular components, the actin cytoskeleton had the most enriched differential genes. The C27 vs. D18 group had the most enriched GO terms, with 46 differential genes annotated. The most enriched differential genes of the C27 vs. D18 group among the cellular components were in the term actin cytoskeleton; among the molecular functions, oxidoreductase activity was the most enriched in differential genes, with 47 differential genes annotated, followed by symporter activity, with 12 differential genes annotated (Figure 5C).

These results provide a detailed overview of the functional categories impacted by temperature stress, highlighting specific biological processes, cellular components, and molecular functions that were significantly altered under different temperature conditions. Comparing changes in organisms under temperature stress can provide us with a better understanding of their vulnerability and adaptation in ecosystems and provide us with new technological tools for ecological engineering to meet the challenges of climate change.

### 3.4. KEGG Enrichment Analysis

**Figure 6:**
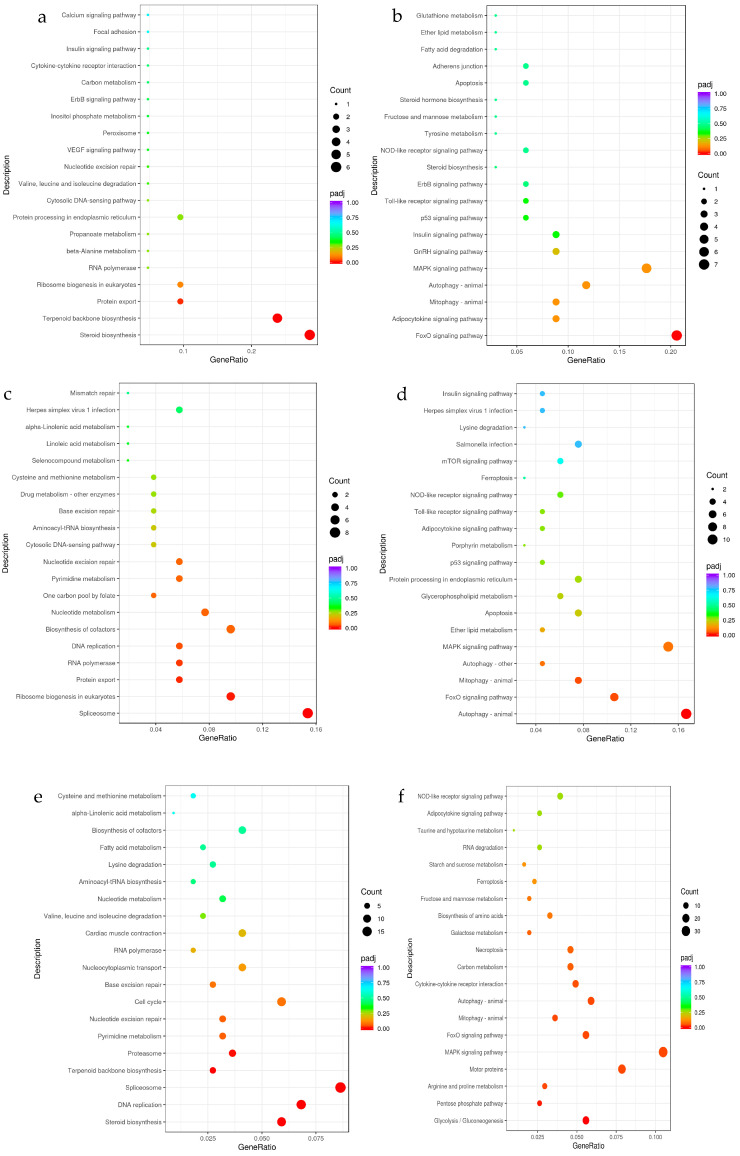
KEGG enrichment analysis of transcriptome differentially expressed genes. (**a**,**b**): KEGG pathway map of down-regulated and up-regulated genes of A21 vs. D18; (**c**,**d**): KEGG pathway map of down-regulated and up-regulated genes of B24 vs. D18; (**e**,**f**): KEGG pathway map of down-regulated and up-regulated genes of C27 vs. D18.

The KEGG database annotates the functions of gene products and their involvement in various metabolic pathways. The KEGG pathway enrichment results for DEGs across temperature treatment groups are illustrated in Figure 6.

The results reveal that compared to the control group, down-regulated genes in the 21 °C experimental group were significantly enriched in pathways related to steroid biosynthesis, terpenoid backbone biosynthesis, and protein export (Figure 6a). Conversely, up-regulated genes were notably enriched in the FoxO signaling pathway (Figure 6b).

In the 24 °C experimental group, down-regulated genes were significantly enriched in pathways associated with the spliceosome, ribosome biogenesis in eukaryotes, protein export, RNA polymerase, and DNA replication (Figure 6c). Meanwhile, up-regulated genes showed significant enrichment in pathways related to autophagy (animal), the FoxO signaling pathway, and mitophagy (animal) (Figure 6d).

In the 27 °C experimental group, down-regulated genes were significantly enriched in pathways associated with steroid biosynthesis, DNA replication, spliceosome, terpenoid backbone biosynthesis, and proteasome (Figure 6e). In contrast, up-regulated genes were prominently enriched in pathways involved in glycolysis/gluconeogenesis, the pentose phosphate pathway, arginine and proline metabolism, motor proteins, the MAPK signaling pathway, the FoxO signaling pathway, mitophagy (animal), autophagy (animal), and cytokine–cytokine receptor interaction (Figure 6f).

These findings underscore the significant pathways and processes affected by temperature stress, highlighting distinct biological and metabolic responses across different temperature conditions.

### 3.5. Real-Time Fluorescence Quantitative PCR Results

**Figure 7:**
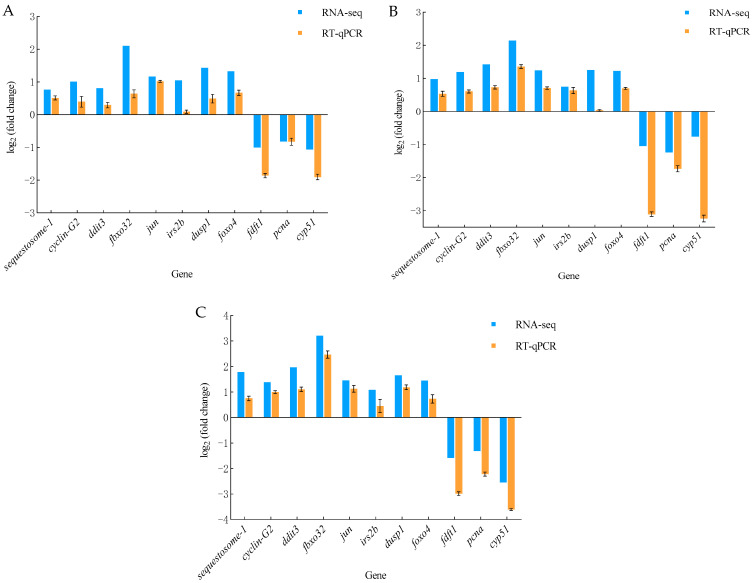
Validation of the DEGs by RT-qPCR. (**A**): Real-Time Fluorescence Quantitative PCR Results at 21 °C; (**B**): Real-Time Fluorescence Quantitative PCR Results at 24 °C; (**C**): Real-Time Fluorescence Quantitative PCR Results at 27 °C.

To validate the accuracy of the RNA-seq results, 11 DEGs with significant changes were selected from the transcriptomic analysis. These genes were subjected to quantitative PCR (RT-qPCR) for relative quantification to assess the differences in gene expression between the temperature treatment groups and the control group. The results demonstrate that the overall expression trends observed in the RT-qPCR analysis are consistent with those identified in the RNA-seq data (Figure 7). This concordance confirms the reliability of the transcriptomic sequencing results obtained in this study.

## 4. Discussion

With global warming and an increase in extreme weather events, serious challenges to the survival and reproduction of living organisms have emerged. We found that the optimal incubation temperature for expanded seahorses is about 18 °C, so we used 18 °C as a control group. Martinez-Cardenas et al. found that expanded seahorse larvae could survive for a short period of time at 26 °C in their study on the effect of temperature on the growth and survival of expanded seahorse larvae in the early stage of culture [12]. In our previous experiments related to temperature stress, we found that seahorse larvae were unaffected by 21 °C and 24 °C and were able to survive for a shorter period of time during the 24 h incubation. By comparing the mortality rate at different temperatures, we found that the 24 h upper incipient lethal temperature was between 27 and 28 °C, so 27 °C was taken as the extreme temperature that could be tolerated in the short term.

Transcriptome sequencing has proven to be a powerful tool for identifying DEGs, annotating gene functions, analyzing related metabolic pathways, and understanding the adaptive mechanisms of organisms under environmental stress [13,14]. In the face of climate change, organisms respond to environmental stresses by adjusting their transcriptome to maintain physiological homeostasis and adapting to new environmental conditions by up- or down-regulating the expression levels of specific genes and changes in KEGG-related pathways. Yang et al. utilized transcriptome sequencing to investigate the effects of high-temperature stress on pearl gentian grouper (*Epinephelus lanceolatus*) and found that the number of up-regulated genes exceeded that of down-regulated genes in comparisons between different temperature groups [15]. This suggests that the observed changes in DEGs may accelerate thermal damage to pearl gentian grouper. Similarly, Deng et al. sequenced yellow croaker (*Larimichthys crocea*) under high-temperature stress and identified 1259 DEGs compared to the control group, with 821 genes up-regulated and 438 genes down-regulated [16]. They hypothesized that high temperatures activate cellular metabolism to mitigate the damage caused by heat stress.

In this study, we compared the DEGs of *H. abdominalis* larvae cultured under different temperatures with those of the control group, identifying a total of 1675 DEGs. Consistent with the findings in pearl gentian grouper and yellow croaker studies, the number of up-regulated genes was higher than that of down-regulated genes across all comparisons. This indicated that temperature stress induced significant changes in gene expression in seahorse larvae, with higher temperatures resulting in increased up-regulation of DEGs as a response to thermal stress. Furthermore, a total of 78 common DEGs were identified across all temperature treatment groups compared to the control group. This suggested that these genes played a crucial regulatory role in the response of the seahorse larvae to high-temperature stress.

Heat shock proteins (HSPs), also known as stress proteins, are a class of proteins synthesized when cells are exposed to external environmental stress. They protect cells from environmental extremes by enhancing their stress capacity and have, therefore, attracted extensive research attention in aquatic animal studies. HSP maintains homeostasis by promoting the synthesis of stress proteins in response to sudden changes in the environment. Fish such as *Hippocampus erectus* and yellow croaker both showed significant increases in the expression levels of the *HSP* gene family following temperature stress [17,18]. In this study, we found that the expression of HSP40, HSP70, and HSP90 protein families were significantly increased in the significant differential genes. And more types of genes were identified as the temperature increased.

In the context of global climate change, organisms are facing unprecedented environmental pressure, and biodiversity conservation is particularly important. By combining the results of GO enrichment analysis of DEGs with climate change factors, it is possible to explore how organisms can respond to the challenges of climate change through the adjustment of gene expression. This integrated analysis helps us to better understand the mechanism of biodiversity maintenance and the future development trend of ecosystems. Meanwhile, KEGG enrichment analyses provide molecular-level evidence for understanding how species adapt to climate change, which is crucial for developing effective biodiversity conservation strategies. By identifying those species and metabolic pathways that are sensitive to temperature stress, we can prioritize the conservation of these vulnerable species and take appropriate measures to help them adapt to climate change. The results will also help us to assess the impact of climate change on the overall level of biodiversity and develop appropriate response strategies.

GO functional enrichment analysis of the DEGs revealed that as the temperature stress increased for *H. abdominalis* larvae, there was a more pronounced enrichment of GO terms related to metabolic processes, redox reactions, and biosynthesis. These findings are consistent with results from studies on Nile tilapia (*Oreochromis nilotica*) [19], Arctic grayling (*Thymallus arcticus grubei*) [20], and yellow croaker [18], indicating that organisms adjust metabolic processes to mitigate stress-induced damage when exposed to temperature stress. Additionally, to maintain lower levels of reactive oxygen species (ROS) generated by stress, redox processes played a critical role. Various antioxidant enzyme activities were altered to neutralize excessive ROS. This suggested that, in response to temperature stress, seahorse larvae experienced increased energy expenditure and metabolic activity. The DEGs likely interacted through various metabolic pathways and redox-related pathways to collectively manage temperature-induced stress.

When the external environment of an organism changes, its internal genes or signaling pathways undergo alterations, which in turn force the entire regulatory network to adapt, striving to maintain a more stable state [16]. In this study, comparative KEGG enrichment analysis of different temperature treatment groups and the control group in seahorse larvae revealed that several signaling pathways were significantly enriched under temperature stress conditions and played crucial roles. Down-regulated DEGs were notably enriched in pathways involved in steroid biosynthesis, terpenoid backbone biosynthesis, the spliceosome, and DNA replication. Specifically, DNA replication and spliceosome pathways were significantly enriched in B24 vs. D18 and C27 vs. D18 comparisons. Genes involved in these pathways, including the *PCNA* and *Mcm* gene families, as well as *Sm* proteins, showed significant down-regulation. These findings are consistent with the trends observed by Bilyk and Huang et al., indicating that as temperature stress increases, the processes of cell proliferation and protein biosynthesis in seahorse larvae generally slow down [21,22].

After high-temperature stress, up-regulated DEGs in seahorse larvae were significantly enriched in the FoxO signaling pathway and the mitophagy pathway. Notably, the FoxO signaling pathway was prominently enriched across all comparison groups, with differential genes such as *CyclinG2* and the *FoxO* family members showing increased expression. *CyclinG2*, a protein-coding gene, plays a role in halting cell cycle progression, and overexpression of *CyclinG2* can induce cell cycle arrest [23,24]. As a transcriptional activator, *FoxO* influences various biological functions by regulating downstream target genes. It is involved in DNA damage repair, oxidative stress response, apoptosis, autophagy, cell proliferation and differentiation, and cellular metabolism [25,26,27]. These findings indicate that, under temperature stress, seahorse larvae regulate the cell cycle and apoptosis through changes in genes within the FoxO signaling pathway, which may contribute to increased mortality observed during stress conditions.

In the mitophagy pathway, significantly up-regulated genes include *c-jun* and the *HIF1* family, with *BNIP3* and *NBR1* also showing notable up-regulation as stress temperatures increase. The gene *c-jun* acts as a downstream regulatory factor in the nuclear factor-κB (NF-κB) signaling pathway. It can be either up-regulated or down-regulated in response to oxidative stress, thereby influencing various biological processes such as cell proliferation and apoptosis [28,29]. The up-regulation of *c-jun* following temperature stress may be a mechanism to counteract cell death caused by high temperatures in seahorse larvae. Increased expression levels of *HIF1* activate the downstream gene *BNIP3*. Research indicates that the *HIF*-induced pro-apoptotic gene *BNIP3* is associated with cell death [30]. This suggests that higher stress temperatures lead to the up-regulation of numerous apoptosis-related genes, contributing to cellular damage in seahorse larvae. Additionally, *NBR1*, essential for peroxisome turnover and involved in endogenous peroxisome conversion, is also up-regulated [31]. Changes in *NBR1* are generally related to antioxidant enzyme levels. Its up-regulation can result in decreased levels of catalase, further illustrating how high temperatures can disrupt the antioxidant system in fish.

## 5. Conclusions

Global warming-induced heat stress is increasingly becoming a key factor threatening the survival of aquatic organisms, and temperature, as a key parameter in the external environment of organisms, has a profound impact on the physiological functions, behavioral habits, and population dynamics of seahorses. This study employed RNA-seq technology to investigate the effects of acute temperature stress on *H. abdominalis* larvae. The findings revealed a significant increase in the number of differentially expressed genes as stress temperatures rose, with these genes primarily associated with metabolic processes, redox reactions, and biosynthesis. These results underscore the complex impact of temperature stress on *H. abdominalis* larvae and provide essential data for understanding their adaptive mechanisms. Research on the temperature response of the seahorse remains limited. We expect to subsequently refine the thermotolerance experiments and establish finer temperature gradients, such as warming experiments at 0.5 °C or shorter intervals, in order to more accurately determine the hemi-lethal temperature, limiting temperature, and the magnitude of thermotolerance of seahorses. The long-term adaptive changes of expanded-bellied seahorses under different temperature conditions, including the growth rate, survival rate, and physiological indexes, were observed by extending the experimental period. The physiological mechanisms were explored more deeply to study the effects of temperature on the metabolism, enzyme activity, gene expression, and other physiological processes of the expanded-bellied seahorse, so as to reveal the intrinsic mechanisms of its adaptation to temperature changes.

## Figures and Tables

**Table 1:** Primers used in the experiments.

Differentially Expressed Genes	Forward Primer Sequence (5′–3′)	Reverse Primer Sequence (5′–3′)	Amplified Length (bp)	Annealing Temperature (°C)
*sequestosome-1*	GATTCTACGACGACGAACGG	TTTGCCCAGCAGGTAAGC	133	55
*cyclin-G2*	TTGGAAATAGCCCGTCAC	TCATCCCAGCAGCACTCG	230	55
*ddit3*	GCTATCACCCATTCCCACT	CATACCACGCCTCCAACT	279	55
*fbxo32*	CAGGAAGTGGGAGAAGTT	ACGGCAAGAAGTCAAGTA	174	55
*jun*	CTGACTCGCCTGTTTACG	TGGGAAGAGGAGGTGGAT	163	55
*irs2b*	CCCCAACGGACTCAACTAC	TGAAGCCCAAGCAAACAT	137	55
*dusp1*	ACTGTCGCTCTTTCCTATCC	AAGTCCCGTCTTTCTTGG	222	55
*foxo4*	CGTGGACTACATCATCAACAG	AATGGAGGAAGAGGCAGAG	298	55
*fdft1*	CACAGTATGGATTTGGGAGA	AACCGTGAACAGGGATTT	290	55
*pcna*	CTGTAGTCTCCCGTGTAGC	ATGCCTCCGTGATTAGAT	263	55
*cyp51*	CATCTGGTCCACATTGCT	CACAGGATCGGCTCGTAA	241	55
*ap4b1*	TCTCAGACGCCCTTACCT	CCACAGATTCTCAAACCCT	155	55

**Table 2:** Transcriptome sequencing data statistics of heat-stressed *H. abdominalis* larvae.

Sample	Raw Bases (G)	Clean Bases (G)	Error Rate (%)	Q20 (%)	Q30 (%)	GC (%)
A21-1	6.35	5.93	0.02	98.11	96.47	52.32
A21-2	6.52	5.92	0.02	98.21	96.66	52.91
A21-3	6.80	6.39	0.02	98.30	96.62	53.35
B24-1	6.47	6.16	0.02	98.19	96.34	51.87
B24-2	6.77	6.37	0.02	98.17	96.51	53.16
B24-3	6.19	5.79	0.02	98.23	96.61	53.25
C27-1	6.92	6.45	0.02	98.15	96.49	53.20
C27-2	7.25	6.75	0.02	98.24	96.69	52.65
C27-3	6.92	6.55	0.02	98.13	96.44	52.51
D18-1	6.96	6.41	0.02	98.18	96.57	52.06
D18-2	6.94	6.49	0.02	98.21	96.41	51.56
D18-3	6.87	6.42	0.02	98.38	96.46	51.29

**Table 3:** Transcriptome sequencing data and genome comparison of *H. abdominalis* larvae.

Sample	Total Reads	Total Map	Unique Map	MULTI MAP
A21-1	39,522,906	35,741,630 (90.43%)	34,030,508 (86.10%)	1,711,122 (4.33%)
A21-2	39,495,652	36,563,775 (92.58%)	34,747,355 (87.98%)	1,816,420 (4.60%)
A21-3	42,595,172	38,909,260 (91.35%)	36,925,817 (86.69%)	1,983,443 (4.66%)
B24-1	41,076,774	37,305,596 (90.82%)	35,314,592 (85.97%)	1,991,004 (4.85%)
B24-2	42,468,594	39,150,508 (92.19%)	37,209,326 (87.62%)	1,941,182 (4.57%)
B24-3	38,581,618	35,541,904 (92.12%)	33,568,929 (87.01%)	1,972,975 (5.11%)
C27-1	42,973,976	39,843,386 (92.72%)	37,734,076 (87.81%)	2,109,310 (4.91%)
C27-2	44,986,444	41,277,431 (91.76%)	39,002,262 (86.70%)	2,275,169 (5.06%)
C27-3	43,647,230	40,331,388 (92.40%)	38,000,786 (87.06%)	2,330,602 (5.34%)
D18-1	42,729,370	39,206,413 (91.76%)	36,909,079 (86.38%)	2,297,334 (5.38%)
D18-2	43,289,626	38,184,351 (88.21%)	36,290,236 (83.83%)	1,894,115 (4.38%)
D18-3	42,780,498	38,476,968 (89.94%)	36,470,173 (85.25%)	2,006,795 (4.69%)

**Table 4:** Sequencing data and statistical distribution of genomic regions in *H. abdominalis* larvae.

Sample	Exon	Intron	Intergenic
A21-1	4,459,275,023 (83.70%)	231,829,881 (4.35%)	636,605,765 (11.95%)
A21-2	4,616,877,493 (84.65%)	207,458,093 (3.80%)	629,610,344 (11.54%)
A21-3	4,930,294,140 (84.93%)	195,471,967 (3.37%)	679,379,047 (11.70%)
B24-1	4,611,514,520 (82.87%)	201,496,249 (3.62%)	751,744,739 (13.51%)
B24-2	4,986,874,869 (85.40%)	180,454,218 (3.09%)	672,152,429 (11.51%)
B24-3	4,492,933,276 (84.74%)	167,665,253 (3.16%)	641,209,683 (12.09%)
C27-1	4,977,719,258 (83.75%)	190,662,245 (3.21%)	775,051,432 (13.04%)
C27-2	5,100,358,883 (82.86%)	213,417,513 (3.47%)	841,764,754 (13.67%)
C27-3	4,983,132,473 (82.82%)	219,867,981 (3.65%)	814,149,096 (13.53%)
D18-1	4,884,014,252 (83.54%)	221,703,904 (3.79%)	740,588,148 (12.67%)
D18-2	4,776,421,053 (83.87%)	246,803,023 (4.33%)	671,942,060 (11.80%)
D18-3	4,744,164,998 (82.63%)	245,979,219 (4.28%)	751,205,096 (13.08%)

## Data Availability

The data supporting the findings of this study are available within the article and its Supplementary Materials. All relevant data, including datasets analyzed or generated during this study, are included in the submitted manuscript. No additional external datasets were used, and no data are withheld due to privacy or ethical restrictions.

## Supplementary Materials

The following supporting information can be downloaded at https://www.mdpi.com/article/10.3390/genes15101345/s1, Excel S1: Volcano map of differentially expressed genes in *Hippocampus abdominalis* larvae A21vsD18_deg_all.xls. Excel S2: Volcano map of differentially expressed genes in *Hippocampus abdominalis* larvae B24vsD18_deg_all.xls. Excel S3: Volcano map of differentially expressed genes in *Hippocampus abdominalis* larvae C27vsD18_deg_all.xls. Excel S4: Clustering heat map of differentially expressed genes.xls. Excel S5: Principal component analysis diagram.xls. Excel S6: Venn diagram of differentially expressed genes in the transcriptome.xls. Excel S7: GO enrichment analysis of transcriptome differentially expressed genes A21vsD18.all_GOenrich.xls. Excel S8: GO enrichment analysis of transcriptome differentially expressed genes B24vsD18.all_GOenrich.xls. Excel S9: GO enrichment analysis of transcriptome differentially expressed genes C27vsD18.all_GOenrich.xls. Excel S10: KEGG enrichment analysis of transcriptome differentially expressed genes A21vsD18.down_KEGGenrich.xls. Excel S11: KEGG enrichment analysis of transcriptome differentially expressed genes A21vsD18.up_KEGGenrich.xls. Excel S12: KEGG enrichment analysis of transcriptome differentially expressed genes B24vsD18.down_KEGGenrich.xls. Excel S13: KEGG enrichment analysis of transcriptome differentially expressed genes B24vsD18.up_KEGGenrich.xls. Excel S14: KEGG enrichment analysis of transcriptome differentially expressed genes C27vsD18.down_KEGGenrich.xls. Excel S15: KEGG enrichment analysis of transcriptome differentially expressed genes C27vsD18.up_KEGGenrich.xls. Excel S16. Validation of the DEGs by RT-qPCR.
